# Age-Related Differences in Hearing Function and Cochlear Morphology between Male and Female Fischer 344 Rats

**DOI:** 10.3389/fnagi.2017.00428

**Published:** 2018-01-04

**Authors:** Zuzana Balogová, Jiří Popelář, Francesca Chiumenti, Tetyana Chumak, Jana Svobodová Burianová, Natalia Rybalko, Josef Syka

**Affiliations:** ^1^Institute of Experimental Medicine of the Czech Academy of Sciences, Prague, Czechia; ^2^Faculty of Medicine, University of Padova, Padova, Italy

**Keywords:** Fischer 344 rats, aging, gender differences, hearing function, cochlear morphology, stria vascularis

## Abstract

Fischer 344 (F344) rats represent a strain that is frequently used as a model for fast aging. In this study, we systematically compare the hearing function during aging in male and female F344 rats, by recording auditory brainstem responses (ABRs) and distortion product otoacoustic emissions (DPOAEs). In addition to this, the functional parameters are correlated with the cochlear histology. The parameters of the hearing function were not different in the young (3-month-old) male and female F344 rats; the gender differences occurred only in adult and aged animals. In 8–24-month-old males, the ABR thresholds were higher and the ABR amplitudes were smaller than those measured in females of the same age. There were no gender differences in the neural adaptation tested by recording ABRs, elicited by a series of clicks with varying inter-click interval (ICI). Amplitudes of DPOAEs in both the males and females decreased with age, but in the males, the decrease of DPOAE amplitudes was faster. In males older than 20 months, the DPOAEs were practically absent, whereas in 20–24-month-old females, the DPOAEs were still measurable. There were no gender differences in the number of surviving outer hair cells (OHC) and the number of inner hair cell ribbon synapses in aged animals. The main difference was found in the stria vascularis (SV). Whereas the SV was well preserved in females up to the age of 24 months, in most of the age-matched males the SV was evidently deteriorated. The results demonstrate more pronounced age-related changes in the cochlear morphology, hearing thresholds, ABR amplitudes and DPOAE amplitudes in F344 males compared with females.

## Introduction

Age-related hearing loss (presbycusis) is the most prevalent sensory disorder in the elderly population (Gates and Mills, 2005; Fetoni et al., 2011). Presbycusis is a complex phenomenon characterized by a combination of the deteriorated function in the periphery, as well as in the central auditory system. In the development of presbycusis mutation of the mitochondrial DNA, other genetic disorders and systemic diseases (hypertension, diabetes, metabolic diseases) may contribute (Gates and Mills, 2005; Huang and Tang, 2010; Fetoni et al., 2011). The state of presbycusis is also influenced by the effects of noise, consumption of ototoxic drugs and diet during the life-time. Pathological processes leading to the occurrence of presbycusis, may be present in different parts of the auditory system. In the inner ear losses are often present: of inner and outer hair cells (OHC), of ribbon synapses between inner hair cells (IHC) and auditory nerve fibers of spiral ganglion neurons- and atrophy of the stria vascularis (SV), resulting in a decline of the endolymphatic potential. The central component of presbycusis, i.e., the pathological processes occuring in neurons of the central auditory system, affect the temporal processing of acoustical stimuli and cause a cognitive decline. This results in deteriorated speech-understanding and particularly in speech-perception difficulties in a noisy environment. Presbycusis is the factor that markedly affects patients’ quality of life and represents a serious social and economic problem (World Health Organization, 2017).

The Fisher 344 (F344) strain is an inbred strain frequently used in the studies of cancer and toxicity. F344 rats develop a fast progressing hearing loss, which is demonstrated by a hearing threshold increase starting at the age of 12 months (Popelar et al., 2006). However, only 10%–20% of the IHC or OHC are missing in aged F344 rats with a severe hearing loss (Popelar et al., 2006; Bielefeld et al., 2008). Similarly, a 29% spiral ganglion cell loss was observed in elderly F344 rats (Buckiova et al., 2006). A serious age-related degeneration of the SV was reported in aging animals with a damaged layer of marginal cells, loss of pigmented cells and reduced vascularity (Gratton and Schulte, 1995; Buckiova et al., 2006; Ohlemiller et al., 2006). These changes in the peripheral hearing organ are accompanied by pathologies in the central auditory system, represented mainly by a significant decline in the glutamic acid decarboxylase (GAD) level and changes in the expression of parvalbumin-immunoreactive neurons in the central auditory system (Burianová et al., 2009; Ouda et al., 2012, 2015). For a review of age-related changes in the auditory system of F344 rats, see Syka (2010).

Most of our previous results were obtained in F344 males. Preliminary experiments performed on F344 females, indicated that females possess better parameters of hearing function than age-matched males. The aim of this study was to characterize the structural and functional differences in the auditory system of male and female F344 rats of different ages, by comparing their auditory brainstem responses (ABRs), distortion products of otoacoustic emissions (DPOAEs), and the changes in cochlear morphology.

## Materials and Methods

### Animals

Hearing function was investigated in male and female F344 rats in five age groups: 3-month-, 8-month-, 12-month-, 22–24-month- and 27–30-month-old animals. The animals in all of the age groups were in good health. HEINE mini 2000 otoscope was used to exclude any eardrum pathology. Fischer 344 rats were obtained from Charles River Deutschland (Sulzfeld, Germany) and subsequently maintained in a local facility through inhouse breeding. All of the animals were housed in age-matched groups of two or three per cage, under standard laboratory conditions, in a constant environment and a 12/12 h normal light/dark cycle; food and water were available *ad libitum*. All experimental procedures were approved by the Ethical Committee of the Institute of Experimental Medicine of the Czech Academy of Sciences, and followed the guidelines of the EU Directive 2010/63/EU for animal experiments.

### Auditory Brainstem Responses (ABRs) Recording

ABRs were recorded in animals placed in a sound proof and anechoic room using acoustical free-field stimulation. The walls and ceiling inside the room were covered by cones from phono-absorbent material. Acoustic measurements demonstrated that the attenuation inside the room against the noise level outside the room, was 55 dB at 250 Hz and 60–70 dB for frequencies above 500 Hz. The acoustic system inside the room was calibrated with a Bruel&Kjaer^®^ 4939 microphone, a ZC0020 preamplifier and a B&K 2231 sound level meter (BRÜEL & KJÆR SOUND & VIBRATION MEASUREMENT A/S, Nærum, Denmark). The frequency–response curve of this system was relatively flat and varied by less than ±9 dB between 0.15 kHz and 40 kHz.

The hearing thresholds in rats were assessed by recording the ABRs in the animals under light anesthesia (ketamine 35 mg/kg, xylazine 6 mg/kg), using subcutaneous needle electrodes placed on the vertex (an active electrode) and in the neck muscles (ground and reference electrodes). The signal from the electrode was amplified 10,000-times using RA16PA RA4LI preamplifier (Tucker Davis Technologies, SystemIII, Alachua, FL, USA), band-pass filtered over the range of frequencies from 300 Hz to 3 kHz, processed with a TDT RX5-2 Pentusa Base Station (Tucker Davis Technologies, SystemIII, Alachua, FL, USA) and analyzed with BioSig software.

ABRs were evoked by tonal stimuli (3-ms duration, 1 ms rise/fall times) in the frequency range from 2 kHz to 40 kHz, or by clicks generated with a RP2.1 enhanced real-time processor (Tucker Davis Technologies, SystemIII, Alachua, FL, USA). To test the neural adaptability, a series of four clicks with variable inter-click interval (ICI) ranging from 1 ms to 50 ms were used. Acoustic stimuli were delivered in free-field conditions via a two-way loudspeaker system [Jamo^®^ 22496 woofer (Jamo Denmark ApS, Niva, Denmark) and SEAS Excel^®^ T25CF 002 tweeter (SEAS FABRIKKER AS, Moss, Norway)], placed 70 cm in front of the animal’s head. The ABR threshold to each frequency was determined as the minimal tone intensity that still evoked a noticeable potential peak in the expected time window of the recorded signal.

### Distortion Product Otoacoustic Emissions (DPOAEs) Recording

To test the functional status of the OHC, the DPOAEs were recorded with a low-noise microphone system (Etymotic probe ER-10B+; Etymotic Research, Elk Grove Village, IL, USA). DP-grams (the function of the DPOAE level on increasing stimulus frequency) were recorded with a resolution of four points per octave over the frequency range from 1 kHz to 38 kHz. During DPOAE recording, in addition to DPOAE amplitude, also the F1 and F2 intensities in the outer ear canal were monitored to confirm the correct placement of the recording probe. Acoustic stimuli (two primary tones with frequency ratio *F*_2_/*F*_1_ = 1.21 and level *L*_1_ = *L*_2_ = 65 dB) were generated by a TDT System III (RP2 processor; sampling rate 100 kHz) and presented to the ear canal with two custom-made piezoelectric stimulators connected to the probe with 10-cm-long silastic tubes. The signal from the microphone was analyzed by a TDT System III (RP2 processor; sampling rate 100 kHz). DPOAEs were measured in both ears of the animals. The recording of DPOAEs were performed in an anechoic and sound proof room.

During ABR and DPOAE recordings, the rats were placed on a heating pad that automatically maintained body temperature at 38°C.

### Cochlear Histology

Deeply anesthetized animals (ketamine 55 mg/kg and xylazine 8 mg/kg i.m.) were sacrificed and cochleas were quickly removed from the skull. Holes were made in the apical turn, round and oval windows, and cochleas were immersed in 4% paraformaldehyde solution for 3 h (whole mount preparation, *n* = 4 per group) or overnight (paraffin sectioning, *n* = 8 in young groups, *n* = 20 in old groups). One ear from each animal was used for cochlear cross-section preparation; the opposite ear was used for cochlear whole mounts in some animals.

For whole mount preparation the bone of the cochlear wall and tectorial membrane were removed gradually from the cochlear apex to the base and basilar membrain together with organ of Corti was microdissected into four pieces per each cochlea. Cochlear whole mounts then were stained with anti-C-terminal binding protein 2 antibodies (anti-CtBP2, BDTransduction Laboratories, San Jose, CA, USA) for presynaptic hair cell ribbons analysis, phalloidin (# 59033, Dyomics) for hair cell visualization, and DAPI to counterstain cell nuclei. Cochlear preparations were visualized using confocal microscope Zeiss LSM 510 Duo. For each analyzed cochlea, low magnification images of all of the whole mount parts were acquired and localization of certain frequency was determined, based on a percentage distance from base using ImageJ Plugin Measure_Line^1^.

The numbers of OHCs and IHC ribbons were counted using ImageJ software (Schneider et al., 2012). Part of the basilar membrane, hosting 60 OHCs (20 in each raw) in the proximity of certain frequency was analyzed. Present and missing cells were counted and the percentage of cells present was calculated. The mean value for each frequency from four cochleas per group was plotted in the resulting graph. IHC ribbon synapses were counted in image stacks. Five adjacent IHC in the proximity of the particular frequency were chosen and the synaptic puncta relating to them were counted, using Cell counter plugin of ImageJ software (Schneider et al., 2012). The number of synaptic puncta per one IHC was calculated for each frequency in each cochlea.

To analyze the SV, cochlear cross-sections were used. The fixed tissues were decalcified in ethylendiaminetetraacetic acid (0.5 M, pH 8.0) for 2 weeks, dehydrated through a graded alcohol series, cleared in toluene, and embedded in parafin. The paraffin blocks were cut into 5-μm horizontal serial sections. The slices were deparaffinized and hydrated. Tissue sections were treated with Bouin’s solution to enhance the final coloration and stained with Weigert’s iron hematoxylin and Masson’s trichrome staining kit (HT15-1KT, Sigma-Aldrich) visualizing collagen. The changes of stria vascularis morphology were analyzed using light microscope Leica DMRXA.

### Statistical Analysis

The differences between ABR hearing thresholds, DPOAE amplitudes, number of OHCs and IHC ribbon synapses in F344 males and females were tested using two-way ANOVA and Bonferroni *post hoc* test. The differences between ABR amplitudes and ABR thresholds at 8 kHz tones were tested using an unpaired *t*-test.

## Results

### ABR Hearing Thresholds

**Figure 1:**
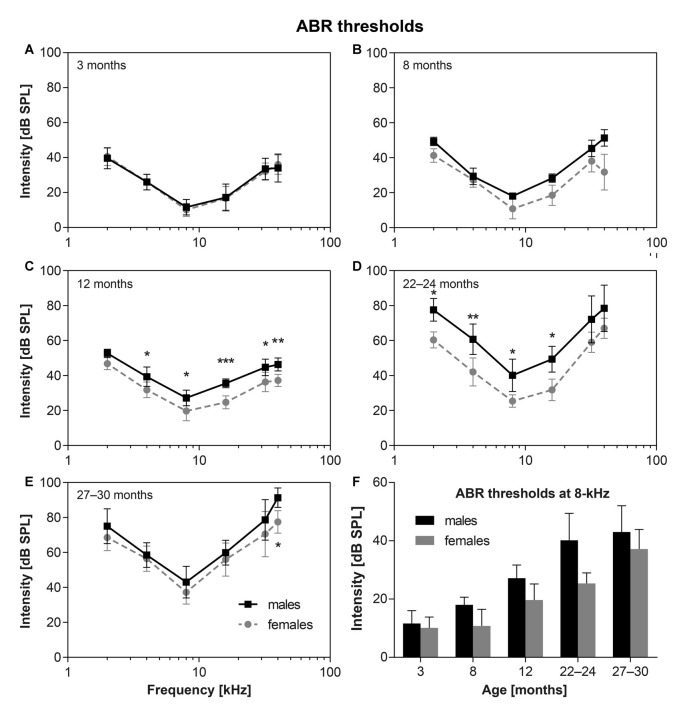
**(A–E)** The average auditory brainstem response (ABR) thresholds in Fischer 344 (F344) males and females of different ages. *p < 0, 05; **p < 0, 01; ***p < 0.001; two-way ANOVA, Bonferroni *post hoc* tests. Mean ± SEM. **(F)** Comparison of ABR thresholds at 8 kHz for F344 males and females of individual age category. Mean ± SEM.

The average ABR hearing thresholds (ABR audiograms) for F344 male and female rats of different ages are shown in Figure 1. The ABR thresholds in young male and female F344 rats (3-months-old) were found to be almost identical (Figure 1A, *n* = 10m + 10f). In the higher age categories the ABR thresholds increased, but the hearing alterations started later in the F344 females and progressed more slowly than in the F344 males. In contrast, the ABR thresholds in females aged 8 months (Figure 1B, *n* = 4m + 4f) were the same as in 3-month-old females, while the ABR thresholds in 8-month-old males increased in comparison with 3-month-old males, and they were significantly higher at 32–40 kHz in comparison with 8-month-old females (*p* < 0.05, Bonferroni *post hoc* test). In 12-month-old males (Figure 1C, *n* = 6m + 6f) the ABR thresholds were significantly higher, in comparison with age-matched females at frequencies 4–32 kHz (*p* < 0.05 and *p* < 0.001, Bonferroni *post hoc* test). In 22–24-month-old males (Figure 1D, *n* = 8m + 6f), the ABR thresholds were significantly higher at frequencies 2–16 kHz in comparison with females (*p* < 0.05 and *p* < 0.001, Bonferroni *post hoc* test). In 27–30-month-old males (Figure 1E, *n* = 10m + 8f), the hearing loss increased more rapidly in females than in males which resulted in a decrease of differences between hearing thresholds in these oldest females and males. Even though the ABR thresholds in F344 males and females were significantly different (*p* < 0.0001, two-way ANOVA), the differences in individual frequencies was significant only at frequency 40 kHz (*p* < 0.05, Bonferroni *post hoc* test).

Figure 1F demonstrates the comparison of the time pattern of ABR threshold changes at 8 kHz for males and females. In males the ABR threshold at 8 kHz increased continuously, and almost linearly, from the age of 3 months to the age of 27–30 months. In females this threshold increase started later (in females older than 8 months) and continued to the age category 22–24 months. However, the ABR threshold at 8 kHz in the F344 females aged 22–24 months started to increase with a faster rate than in males and at the age of 27–30 months, ABR thresholds in females almost reached the thresholds in the age-matched F344 males. Whereas ABR thresholds at 8 kHz in 22–24 months old males and females were significantly different (*p* < 0.0023, unpaired *t*-test), ABR thresholds at 8 kHz in 27–30 month old males and females were not significantly different (*p* < 0.114, unpaired *t*-test; Figure 1F).

### ABR Amplitudes

**Figure 2:**
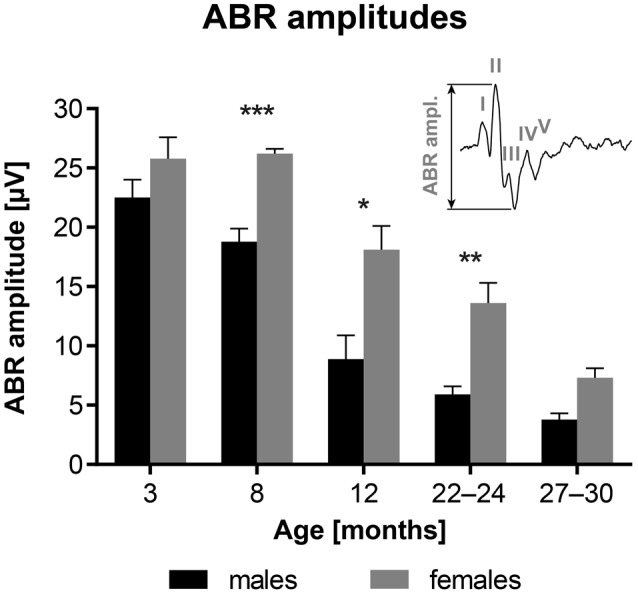
Age-related changes in the average ABR amplitudes in F344 rats, evoked by clicks at 100 dB SPL. *p < 0.05; **p < 0.01; ***p < 0.001; unpaired *t*-test. Mean ± SEM.

Figure 2 shows age-related changes in the ABR amplitudes, evoked by clicks at 100 dB SPL, and measured as the difference between the value of the positive peak II and the negative through between the peaks II and IV (see inserted schema). The results indicate that ABR amplitudes are significantly smaller in F344 males aged 8–24 months than in age-matched females (*p* < 0.05 to *p* < 0.001; unpaired *t*-test).

**Figure 3:**
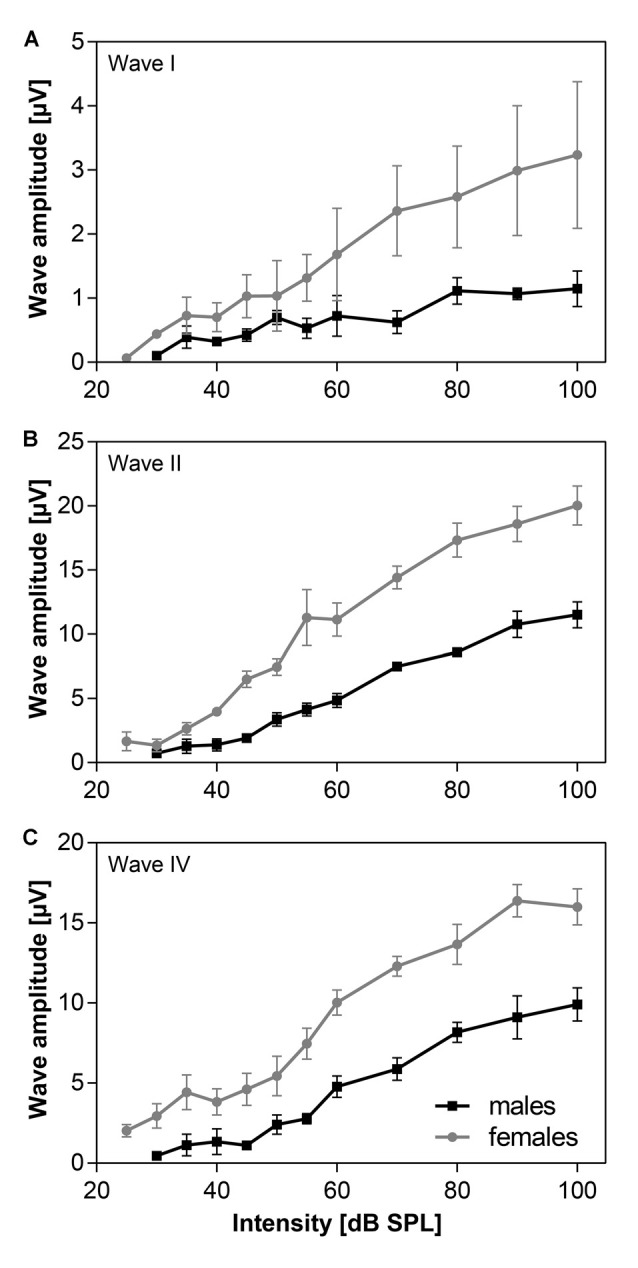
The averaged click-evoked ABR amplitude-intensity functions, *n* = 5m + 5f. **(A)** ABR wave I, **(B)** ABR wave II, **(C)** ABR wave IV. Bars represent mean ± SEM.

Amplitudes of individual ABR waves, evoked by click stimulation of variable intensity, were measured in five males and five females of 24-month old F344 rats. The averaged amplitude-intensity functions for the ABR wave I, II and IV are demonstrated in Figure 3. Maximal wave I amplitude (Figure 3A) in females was three times the maximal wave I amplitude in males. The difference in maximal wave amplitudes is less expressed in later ABR waves. The maximal wave II amplitude (Figure 3B) in females is twice that of males and the maximal wave IV amplitude (Figure 3C) is only 1.6 times that in males. These results indicate that the difference between ABR wave I amplitude, originating at the periphery, in males and females, is larger than the difference between ABR wave IV amplitude, originating in higher auditory structures.

### Neural Adaptation

**Figure 4:**
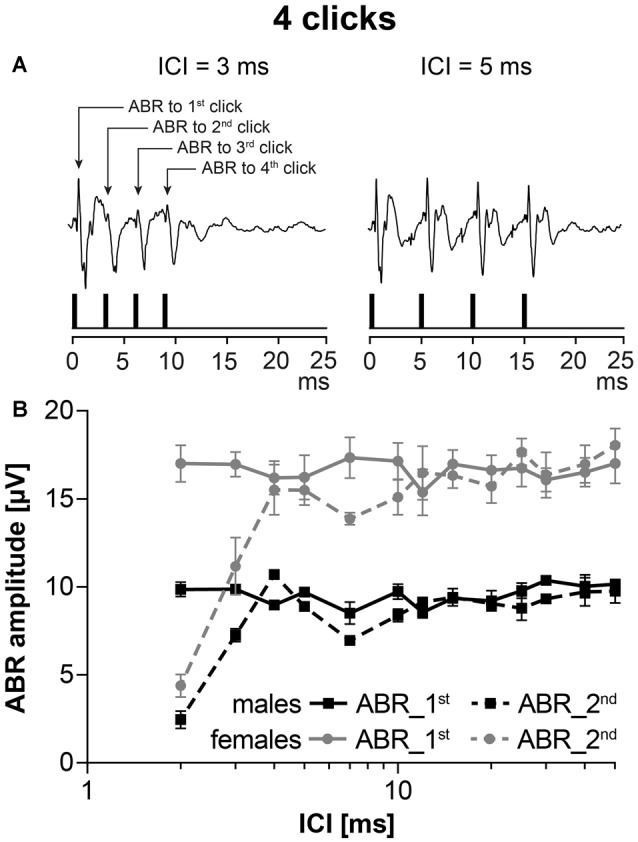
ABRs evoked by stimulation with the series of clicks, intensity 70 dB SPL, with variable interclick interval (ICI). **(A)** Two examples of ABRs to click stimulation with ICI = 3 ms and ICI = 5 ms. **(B)** The averaged ABR amplitude-ICI functions to 1st click stimulation and to 2nd click stimulation in 24-month-old F344 males and females. Bars represent mean ± SEM.

To test the neural adaptation in five males and five females of 24-month old F344 rats, stimulation with a series of four clicks, intensity 70 dB SPL, with variable interclick interval (ICI) was used. Figure 4A demonstrates the schema of an experiment. When the ICI was short (ICI = 3 ms), ABR amplitudes to 2nd, 3rd and 4th clicks were smaller than ABR amplitude to 1st click stimulation. When the ICI was longer (ICI = 5 ms), ABR amplitudes to 2nd, 3rd and 4th click were the same as ABR amplitudes to 1st click stimulation. The function of ABR amplitudes on ICI intervals in aged males and females is demonstrated in Figure 4B. As the ABR amplitudes to the 2nd, 3rd and 4th click stimulation within a particular click series were similar, Figure 4B compares the ABR amplitudes to the 1st and 2nd click stimulation in the F344 males and females. Regardless of the smaller click-evoked ABR amplitude obtained in males, the function of the ABR amplitude on ICI was similar in both sexes, i.e., the amplitude of ABR evoked by the second click, reached the ABR amplitude of the first click at ICI of 4 ms.

### DPOAEs

**Figure 5:**
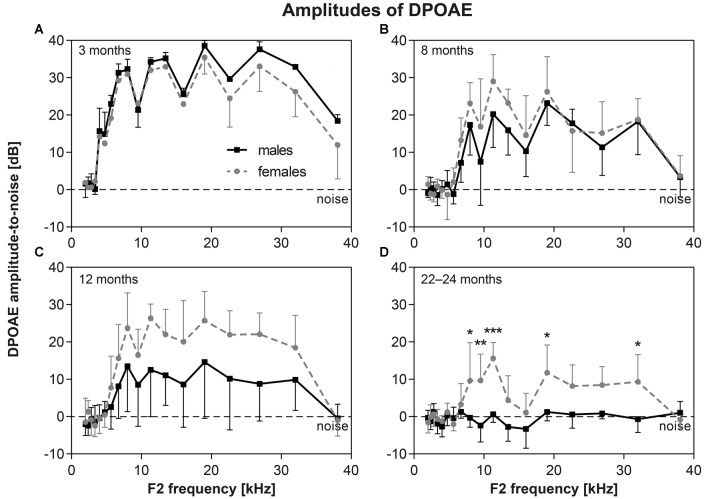
**(A–D)** DP-grams in F344 rats (distortion products of otoacoustic emissions (DPOAE) amplitudes above background noise level) measured in males (*n* = 6–8) and females (*n* = 6–8) of different age categories. *p < 0.05; **p < 0.01; ***p < 0.001; two-way ANOVA, Bonferroni *post hoc* tests. Mean ± SEM.

The functional status of cochlear OHC were checked by the recording of DPOAEs. The average DP grams (i.e., the function of DPOAE amplitudes above background noise level on F2 stimulus frequency) in six to eight F344 males and females of different age category are present in Figure 5. The DPOAE amplitudes of both males and females is maximal in young, 3-month-old animals (Figure 5A). At a higher age the DPOAE amplitudes of both males and females decreased. In animals aged 12 months (Figure 5C), the DPOAE amplitudes in F344 males were significantly smaller than those in F344 females (*p* < 0.0001, two-way ANOVA), but Bonferroni *post hoc* test did not show any significant difference at individual F2 frequencies. In males older than 20 months, the DPOAEs were practically absent, whereas in some of the 22–24-month-old females, the DPOAEs were still measurable (Figure 5D). In the age category 27–30 months no DPOAE were detectable in either male or female F344 rats (not presented).

### Cochlear Morphology

**Figure 6:**
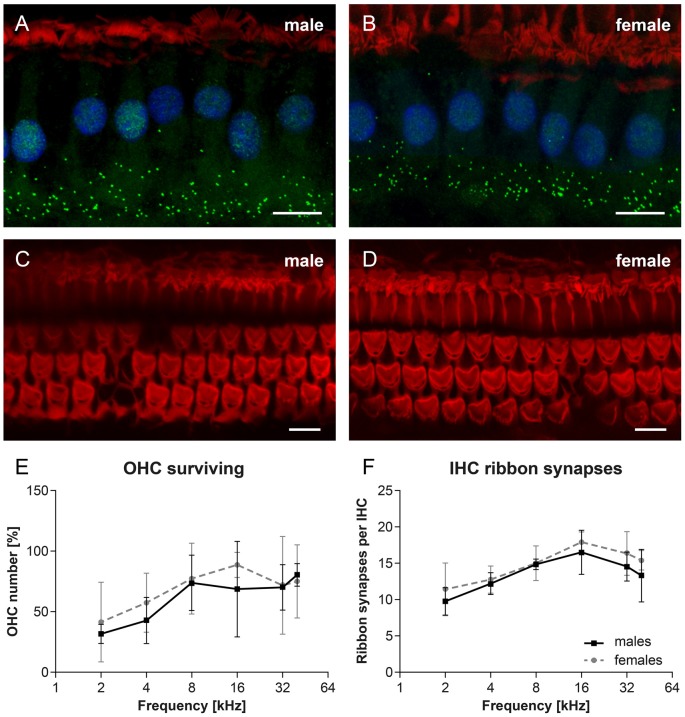
Examples of cochlear whole mounts from 27-month-old rats, showing inner hair cell (IHC) ribbon synapses immunostained with CtBP2 protein (green), in F344 males **(A)** and females **(B)**, and IHCs and OHCs stained with phalloidin (red) in F344 males **(C)** and females **(D)**. Cell nuclei are counterstained with DAPI (blue). **(E)** The average number of surviving OHCs in individual tonotopical regions of the organ of Corti in F344 males and females. **(F)** The average number of IHC ribbon synapses per one IHC localized in individual tonotopical regions of the organ of Corti in F344 males and females. Bars represent mean ± SD. Scale bar = 10 μm.

Cochlear whole mount preparations were used to calculate the number of OHCs and the number of ribbon synapses docking vesicles with neurotransmitter glutamate in the IHCs in 27-month-old F344 males (*n* = 4) and females (*n* = 4). In Figure 6, examples of cochlear whole mounts from aged rats, showing IHC ribbon synapses immunostained with CtBP2 protein (green), in males (Figure 6A) and females (Figure 6B), and IHCs and OHCs stained with phalloidin (red) in males (Figure 6C) and females (Figure 6D), are presented. The average number of surviving OHCs in individual tonotopical regions of the organ of Corti are presented in Figure 6E. The pronounced OHC loss was observed mainly at the apical, low-frequency end of the cochlea (50%–60%), whereas only a few OHCs were missing at the basal, high-frequency part of the cochlea (less than 10%). However, the difference between both curves is not statistically significant (*p* = 0.364; two-way ANOVA). The quantitative analysis of the number of IHC ribbon synapses per one IHC (Figure 6F) demonstrated that the average number of ribbon synapses per IHC is almost identical in both F344 males and females (*p* = 0.071; two-way ANOVA).

#### Stria Vascularis

**Figure 7:**
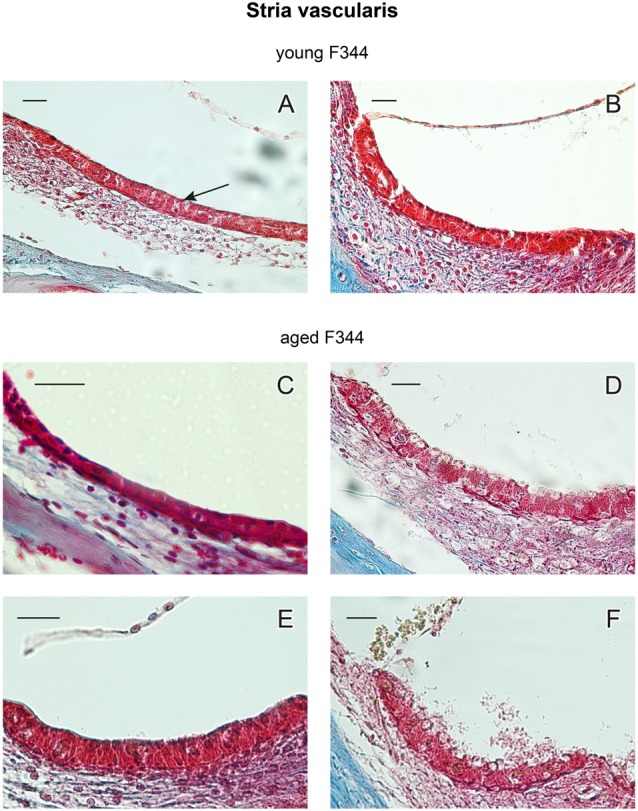
Examples of cross-sections of the stria vascularis (SV) in young **(A,B)** and 27-month-old F344 females **(C,E)** and males **(D,F)**. The arrow in **(A)** marks the marginal layer of the SV. Scale bar = 25 μm.

The main structural difference between males and females has been found in the morphology of the marginal layer of the SV. Single-cell marginal layer of the SV (marked by an arrow in Figure 7A) consists of marginal cells located on the surface of the SV. The examples of histological section of SV in young (4 month old) and aged (27-month-old) rats are presented in Figure 7. Most of SV samples were taken from the middle cochlear turn. SV in young animals (Figures 7A,B, *n* = 4m + 4f) is well preserved, the layer of the marginal cells is covered by a thin layer of collagen fibers. SV in aged female rats, presented in Figures 7C,E, is also well preserved with a continuous layer of marginal cells. In contrast to the old F344 females, the SV in old males (Figures 7D,F) shows many degenerative changes, mainly in marginal cells that are destroyed and vacuolized.

Degenerative changes in the marginal layer of the SV in aged males were usually present in the whole extent of the cochlea. However, in two males (out of 10), the SV was damaged in only several parts of the cochlear coils, whereas in other parts of the cochlea, the SV degenerative changes were less evident. In the other hand, in three aged F344 females (out of 10) the signs of degenerative changes were present in isolated parts of the cochlea. Complete degenerative SV changes were observed in 80% of aged males and partial degeneration was found in 20% of aged males. The marginal layer of the SV was fully preserved in 70% of aged females, whereas in 30% of aged females the signs of degenerative changes in isolated parts of the cochlea were observed.

## Discussion

The results of this study demonstrate the different pattern of the gradual deterioration of the hearing function during aging in F344 males and females. The hearing alterations started later in F344 females (after 8th month of age) than in F344 males (after 3rd month of age) which resulted in the ABR threshold differences at most of frequencies between F344 males and females at 12 months and 22–24 months age categories. However, in age category 27–30 months the ABR threshold in the females increased with a faster rate than in males and almost reached the ABR thresholds in the age-matched males.

Similar gender differences were observed in DPOAEs. The decrease of DPOAE amplitudes during aging was faster in males than in females. In males older than 20 months, the DPOAEs were practically absent, whereas in some of the 22–24-month-old females, the DPOAEs were still measurable. In the oldest group of animals (27–30 months old), no DPOAEs were measurable in any males or females.

DPOAEs reflect *in vivo* OHC motility. However, morphological analysis of the inner ear structures demonstrated that the majority of OHCs were present and morphologically intact in 24-month-old F344 females and males, although dysfunctional. Under normal conditions the proper function of the cochlear hair cells is satisfied by the SV, the epithelial structure on the lateral wall of the cochlear duct. SV produces endolymph for the scala media, it is involved in the potassium transport to the endolymphatic space and generates positive endocochlear potential (EP; +80 to 100 mV). In transgenic mice with serious morphological defects of stria such as homozygous transgenic mice with microphthalmia-associated transcription factor gene mutation (Mitf), expressed by missing intermediate cells in the SV, the EP and ABRs were markedly reduced or almost absent (Liu et al., 2016). As previous results demonstrated normal EP in F344 rats (Bielefeld et al., 2008), we do not expect serious morphological alterations of intermediate cells in the SV. Instead, we observed pathological changes of marginal cells, that can be caused by a marked decrease of collagen fibers in the SV and spiral ligament, as previously demonstrated in F344 rats by Buckiova et al. (2006). However, the reason for evident degenerative changes in the SV in aged F344 males, in comparison with age-matched females, are not known. Chen et al. (2009) demonstrated in F344 rats the disruption of OHC motor protein prestin that can be responsible for the suppression of DPOAE amplitudes and reduced cochlear sensitivity. However, these authors did not mention the gender of the investigated F344 rats. It is probable that in our aged F344 males, the pathological state of SV marginal cells evokes functional changes resulting in a dysfunction of the auditory sensory epithelium.

Our results have demonstrated that the maximal ABR wave I amplitude, originating from the activity of auditory nerve fibers (Figure 3A) in females, was much larger than the maximal wave I amplitude in males. However, the difference in the maximal amplitudes of later ABR waves (Figures 3B,C), originating in higher auditory structures, was much smaller in males than in females.

Neural adaptation in both sexes, tested by ABR evoked by a series of clicks, was found to be comparable in both sexes. These results confirmed our hypothesis that the major reason for deterioration of the hearing function is not localized in the central part of the auditory system, but in the peripheral part.

It is known that the function of the SV is similar to some extent to the function of the tubular system in the kidney. For example, diuretics like ethacrynic acid or furosemide, which are known to suppress the function of the Henle loop in the kidney, also suppress the function of the SV (Sellick and Johnstone, 1974; Melichar and Syka, 1978). In many strains of rats, including Fischer 344 rats, renal disease occurs earlier in life and to a more severe extent in males than in females (Abrass et al., 1995; Baylis and Corman, 1998). Two recent studies (Kwekel et al., 2013, 2015) showed sex differences in kidney gene expression in Fischer 344 rats during aging.

Sexual dimorphism was described in Fischer 344 rats in several other functions. In males, in contrast to females, a drastically accelerated rate of peripheral retinal degeneration was observed (DiLoreto et al., 1994). The males were found more likely to lose spatial reference memory with aging than females (Bizon et al., 2009).

The reasons for the earlier occurrence of the above presented pathologies in F344 male rats, in comparison with females, are not known. An important role in the sex difference is played by the estrogen receptors alpha (ERα) and beta (ERβ). Both of these subtypes of estrogen receptors have been identified in the cochlea at locations where hearing impulses are transmitted (inner and OHC, spiral ganglion cells) and in areas responsible for inner ear homeostasis (SV and spiral ligament) and there are indications that they have neuroprotective effects (Stenberg et al., 2001; Meltser et al., 2008). On the other hand, hyperandrogenism in patients with polycystic ovary syndrome was demonstrated to produce high-frequency hearing loss (Oghan and Coksuer, 2012). The gender differences in the hearing function of F344 rats may be due to combination of the damaging effect of testosterone, as well as the protective effects of estrogen and progesterone, which continue to be secreted after the cessation of the estrous cycle in aging females (Markham and Juraska, 2002). In our experiments, the prolonged secretion of sex hormones in females may be the reason for the later start of the hearing threshold increase in females, in comparison with males.

Prolactin (PRL) was recently found in the cochlea in the aged female mice (Marano et al., 2012). Histological sectioning of the cochlea from the aged female mice, followed by immunostaining with antibodies specific to PRL, revealed that spiral ganglion cells and marginal cells of the SV are the sole sites of PRL expression (Marano et al., 2013).

It has previously been shown that prolactin treatment in guinea pigs results in hyperprolactinemia, producing bone dysmorphology of the otic capsule and hearing loss (Horner et al., 2007; Seriwatanachai et al., 2008). Hyperprolactinemia, leading to hearing loss, has been also detected in menopausal women taking hormone replacement therapy containing prolactin (Metka et al., 1994).

This data provided an indication that PRL expression in aged female mice can be potentially linked to abnormal bone metabolism or degeneration of the cochlear structures leading to an age-related hearing loss. In the present study, the hearing threshold in very old F344 females (aged 27–30 months) demonstrates an accelerated increase in comparison with younger females, that can be produced by increased PRL expression in the cochlea in these very old females. Such an accelerated hearing loss was not observed in age-matched males where no PRL expresion was previously observed.

In conclusion, our results have demonstrated the different pattern of the gradual deterioration of the hearing function during aging in F344 males and females. In F344 females the hearing alterations started later in comparison with males which resulted in the ABR threshold and ABR amplitude differences at most of frequencies between F344 males and females at 12 months and 22–24 months of age. In the group of the oldest animals, the differences between the F344 males and females were reduced. The most probable and most evident differences were observed in the morphological deterioration of the SV. Whereas the SV was well preserved in most 24-month-old females, in most aged-matched males the marginal layer of the SV was degenerated and distorted. Another reason for age-related gender differences in the hearing function in F344 rats, could be the different time pattern of the secretion of sex hormones in males and females or the expression of prolactin in very old females. This study presents evidence that the gender of F344 rats should be taken into account when reporting the results of an experimental study.

## Author Contributions

JS and JP designed the study, interpreted the data and wrote the manuscript. ZB, FC, JSB and NR performed recording of the ABR and DPOAE. TC and JSB performed cochlear histology. JP, ZB and JSB performed data processing and statistical analysis.

## Conflict of Interest Statement

The authors declare that the research was conducted in the absence of any commercial or financial relationships that could be construed as a potential conflict of interest.

## Footnotes

^1^http://www.masseyeandear.org/research/otolaryngology/investigators/laboratories/eaton-peabody-laboratories/epl-histology-resources/imagej-plugin-for-cochlear-frequency-mapping-in-whole-mounts
